# Monitoring of Intracellular Tau Aggregation Regulated by OGA/OGT Inhibitors

**DOI:** 10.3390/ijms160920212

**Published:** 2015-08-26

**Authors:** Sungsu Lim, Md. Mamunul Haque, Ghilsoo Nam, Nayeon Ryoo, Hyewhon Rhim, Yun Kyung Kim

**Affiliations:** 1 Center for Neuro-Medicine, Brain Science Institute, Korea Institute of Science and Technology (KIST), Seoul 136-791, South Korea; E-Mails: sungsulim85@gmail.com (S.L.); mamunbge@gmail.com (M.M.H.); gsnam@kist.re.kr (G.N.); 2 Biological Chemistry, University of Science and Technology (UST), Daejeon 305-333, South Korea; 3 Center for Neuroscience, Brain Science Institute, Korea Institute of Science and Technology (KIST), Seoul 136-791, South Korea; E-Mail: ryoo8099@gmail.com; 4 Department of Neuroscience, University of Science and Technology (UST), Daejeon 305-333, South Korea

**Keywords:** tau protein, *O*-GlcNAcylation, *O*-GlcNAc transferase, *O*-GlcNAcase, tau phosphorylation, tau aggregation

## Abstract

Abnormal phosphorylation of tau has been considered as a key pathogenic mechanism inducing tau aggregation in multiple neurodegenerative disorders, collectively called tauopathies. Recent evidence showed that tau phosphorylation sites are protected with *O*-linked β-*N*-acetylglucosamine (*O*-GlcNAc) in normal brain. In pathological condition, tau is de-glycosylated and becomes a substrate for kinases. Despite the importance of *O*-GlcNAcylation in tau pathology, *O*-GlcNAc transferase (OGT), and an enzyme catalyzing *O*-GlcNAc to tau, has not been carefully investigated in the context of tau aggregation. Here, we investigated intracellular tau aggregation regulated by BZX2, an inhibitor of OGT. Upon the inhibition of OGT, tau phosphorylation increased 2.0-fold at Ser199 and 1.5-fold at Ser396, resulting in increased tau aggregation. Moreover, the BZX2 induced tau aggregation was efficiently reduced by the treatment of Thiamet G, an inhibitor of *O*-GlcNAcase (OGA). Our results demonstrated the protective role of OGT in tau aggregation and also suggest the counter-regulatory mechanism of OGA and OGT in tau pathology.

## 1. Introduction

Tau protein is a neuron-specific microtubule-associated protein (MAPs) that stabilizes microtubule and stimulates microtubules assembly [1]. Tau has a microtubule binding domain that contains a number of lysine residues [2]. The positive charges of lysine residues are critical to bind with a microtubule, which is known to be a negatively charged structure [3,4]. When tau is hyperphosphorylated, the charge balance between tau and microtubules is disrupted and tau loses its binding affinity to microtubules. The released tau becomes aggregated into insoluble filaments, called neurofibrillary tangles (NFTs). The accumulation of NFTs in the brain is a pathological hallmark of multiple neurodegenerative diseases including Alzheimer’s disease (AD).

Due to the importance of tau phosphorylation in initiating tau pathology, great effort has been made to control tau phosphorylation [5]. In a healthy neuron, tau phosphorylation is tightly regulated by multiple protein kinases and phosphatases to maintain microtubule dynamics required for neuronal processes. Evidence has shown that tau protein isolated from a healthy brain is also partially phosphorylated with an average of about two moles of phosphate per mole of protein [1,6]. In contrast, tau isolated from the AD patient’s brain contains six to eight moles of phosphate per mole of protein [6]. The hyperphosphorylated tau is released from microtubules and initiates tau pathology. In this regard, inhibiting tau phosphorylation has been considered as the fundamental therapeutic strategy to cure tau-mediated neurodegeneration. However, continuous failures in controlling tau phosphorylation suggest that there might be other regulatory mechanism that has been overlooked [6,7]. Recent data suggest that tau phosphorylation sites are protected with *O*-GlcNAc (*O*-linked β-*N*-acetylglucosamin) in a healthy neuron. The level of *O*-GlcNAcylation is reduced under pathological condition. Then, tau phosphorylation sites are freely exposed to kinases resulting in the generation of hyperphosphorylated tau. Gong’s group firstly found that *O*-GlcNAcylation level in AD brains was 22% lower than that in normal brain, and hyperphosphorylated tau contained four-fold less *O*-GlcNAc than non-hyperphosphorylated tau [8,9]. Vocadlo’s group further demonstrated the reciprocal relationship between phosphorylation and *O*-GlcNAcylation modification of tau by developing an inhibitor of *O*-GlcNAcase, which catalyze the removal of *O*-GlcNAc [10]. The treatment of OGA inhibitor leads to decrease tau phosphorylation by preventing the removal of *O*-GlcNAc from tau. These studies strongly suggest the importance of *O*-GlcNAcylation in controlling tau phosphorylation. There are two enzymes catalyzing the addition and removal of *O*-GlcNAc; *O*-GlcNAc transferase (OGT) and *O*-GlcNAcase (OGA). The pathological role of OGA in promoting tau aggregation has been actively investigated [10,11]. In contrast, the role of OGT in tau pathology has barely investigated so far.

**Scheme 1:**
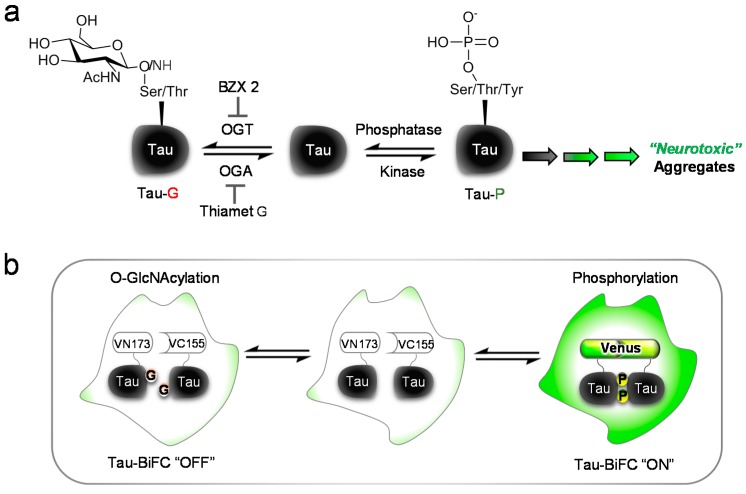
Tau pathology regulated by *O*-GlcNAcylation and phosphorylation: (**a**) A diagram presenting the reciprocal relationship between *O*-GlcNAcylation and phosphorylation in tau pathology. Thiamet G, *O*-GlcNAcase (OGA) inhibitor; BZX2, an *O*-GlcNAc transferase (OGT) inhibitor; Tau-G, *O*-GlcNAcylated Tau; Tau-P, Phosphorylated Tau; and (**b**) Illustration of a cell-based sensor for tau aggregation. In tau-BiFC (bimolecular fluorescence complementation) system, full-length tau is conjugated with the non-fluorescent N- and C-terminal fragment of Venus fluorescence protein. Venus fluorescence turns on only when tau assembles together.

To investigate the role of OGT on tau pathology, here we used a recently developed OGT inhibitor, BZX2, compared with Thiamet G (Scheme 1a) [12]. BZX2 is known to inhibit the activity of purified OGT at the concentration of 15 to 60 µM. To evaluate the compound’s effect on tau aggregation, we treated OGA/OGT inhibitors to tau aggregation sensor cells, named tau-BiFC (bimolecular fluorescence complementation) (Scheme 1b) [13,14]. In tau-BiFC system, non-fluorescent N- and C-terminal compartments of Venus protein are fused to tau, and Venus fluorescence turns on only when tau assembles together. It is beneficial to use tau-BiFC system since there is no fluorescence background when tau expressed in HEK293 exists as a monomer. By eliminating the background noise from monomeric tau, we were able to achieve spatial and temporal resolution of tau dimerization and oligomerization in living cells. By using tau-BiFC sensor, we can monitor and quantify intracellular tau aggregation regulated by BZX2 or Thiamet G.

## 2. Results and Discussion

### 2.1. BZX2 Increases Tau-BiFC (Bimolecular Fluorescence Complementation) Fluorescence Response

**Figure 1:**
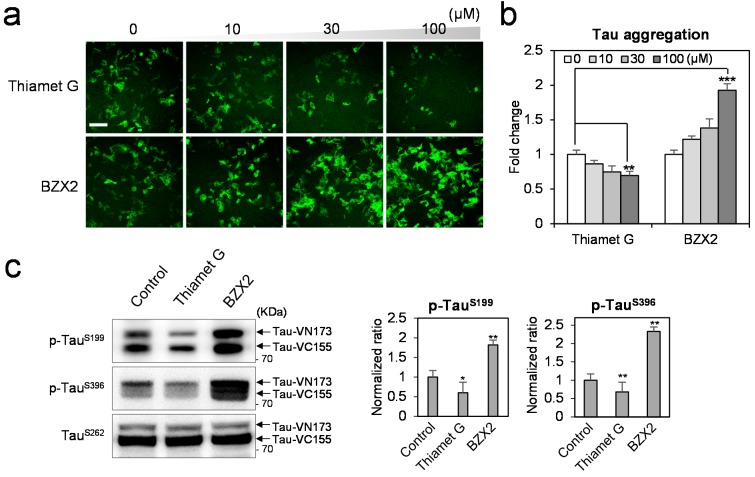

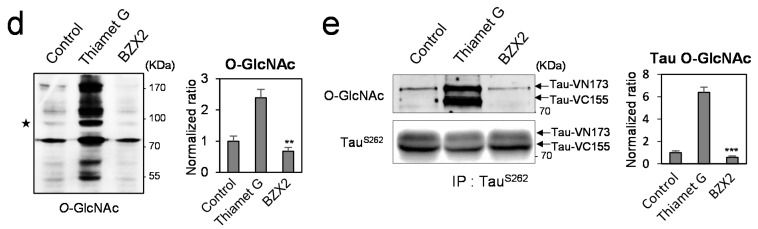
The opposite effects of OGA (*O*-GlcNAcase) and OGT (*O*-GlcNAc transferase) inhibitors on tau pathology. (**a**,**b**) Tau-BiFC cells were treated with Thiamet G or BZX2 (0, 10, 30 and 100 µM) for 24 h. Then, cellular responses of tau-BiFC fluorescence were imaged by using Operetta^®^ (PerkinElmer™, Waltham, MA, USA) (**a**) and quantified by Harmony 3.1 software (PerkinElmer™, Waltham, MA, USA) (**b**). Error bars represent the standard deviations from two independent experiments and each experiment was triplicated. The significance of the experiments was determined with paired *t*-test. ** *p <* 0.01, *** *p <* 0.001. Scale bar = 100 µm; (**c**) Immunoblot analysis of tau phosphorylation and the quantification. For immunoblot analysis, tau-BiFC cells were incubated with Thiamet G (100 µM) or BZX2 (100 µM) for 24 h. The levels of tau phosphorylation were determined using anti-phospho-Ser199 or anti-phospho-Ser396 antibodies. The levels of tau phosphorylation were quantified and normalized with that of non-phosphorylated tau (TauSer262). Black arrows indicate two parts of tau-conjugated BiFC compartments (TauVN173 and Tau-VC155); (**d**) Immunoblot analysis of global *O*-GlcNAc levels in the tau-BiFC cell lysates and the quantification. The star symbol indicates full-length tau; and (**e**) Immunoblot analysis of *O*-GlcNAc levels on immuno-affinity purified tau and the quantification. Immunoblot with anti-tau (TauSer262) antibody indicates the amounts of immune-affinity purified tau. The relative levels of tau *O*-GlcNAcylation were quantified and normalized with that of non-phosphorylated tau (TauSer262). Error bars represent the standard deviations from three independent experiments. The significance of the experiments was determined with paired *t*-test. * *p* < 0.05, ** *p <* 0.01, *** *p <* 0.001.

To compare the effects of OGA and OGT inhibition on tau aggregation, tau-BiFC cells were treated with Thiamet G and BZX2 at various concentrations (Figure 1a). As expected, the treatment of Thiamet G decreased the basal level of tau aggregation slightly by showing 30% reduced tau-BiFC response at the highest concentration (Figure 1b). Conversely, BZX2 treatment increased tau aggregation almost two-fold, showing the strong tau-BiFC fluorescence response (Figure 1b). This result clearly indicates the opposite effect between OGA and OGT inhibitors on tau aggregation. However, OGA and OGT inhibitors do not directly regulate purified tau aggregation *in vitro* without post translational modification on tau (Figure S1). As a pathological indication of tau aggregation, the level of tau phosphorylation was measured by using tau phosphor-antibodies against phospho-Ser199 or phospho-Ser396, which are known for the most sensitive tau phosphorylation sites of the tau-BiFC cell model (Figure 1c) [13]. Upon treatment of BZX2 (100 µM), tau phosphorylation was increased 1.8-fold at Ser199 and 2.3-fold at Ser396 (Figure 1c). The treatment of Thiamet G (100 µM) decreased tau phosphorylation, showing a 40% decrease at Ser199 and 32% decrease at Ser396 (Figure 1c). The opposed level of tau phosphorylation indicates the reciprocal effects of OGA/OGT inhibitors on tau aggregation.

Next, we evaluated intracellular *O*-GlcNAcylation level by using an anti-*O*-GlcNAc antibody (Figure 1d) [8,11]. Upon the treatment of Thiamet G, intracellular *O*-GlcNAcylation level was increased 2.4-fold in total cell lysates. Upon treatment of BZX2, *O*-GlcNAcylation level was decreased by 32% (Figure 1d). This result shows that *O*-GlcNAcylation of intracellular proteins was expository regulated by the treatment of OGA and OGT inhibitors. However, tau is one of the hundreds of protein substrates regulated by OGT and OGA. To scrutinize the level of tau *O*-GlcNAcylation, tau protein was immuno-precipitated with an anti-tau (TauSer262, Abcam, Cambridge, MA, USA) antibody. Then, the purified tau was labeled with an anti-*O*-GlcNAc antibody (Figure 1e). The level of *O*-GlcNAcylated tau was quite low at the basal condition. Upon treatment of Thiamet G, the level of tau *O*-GlcNAcylation was increased by 6.4-fold. Compared to the significantly increased tau *O*-GlcNAcylation, the anti-aggregation effect of Thiamet G was merely 30%. This might be due to the low aggregation propensity of tau under the basal condition. Therefore, the protection effect of Thiamet G needs to be investigated in the condition stimulating tau aggregation.

### 2.2. Counter Regulatory Effect of OGA/OGT Inhibitors on Tau Phosphorylation

**Figure 2:**
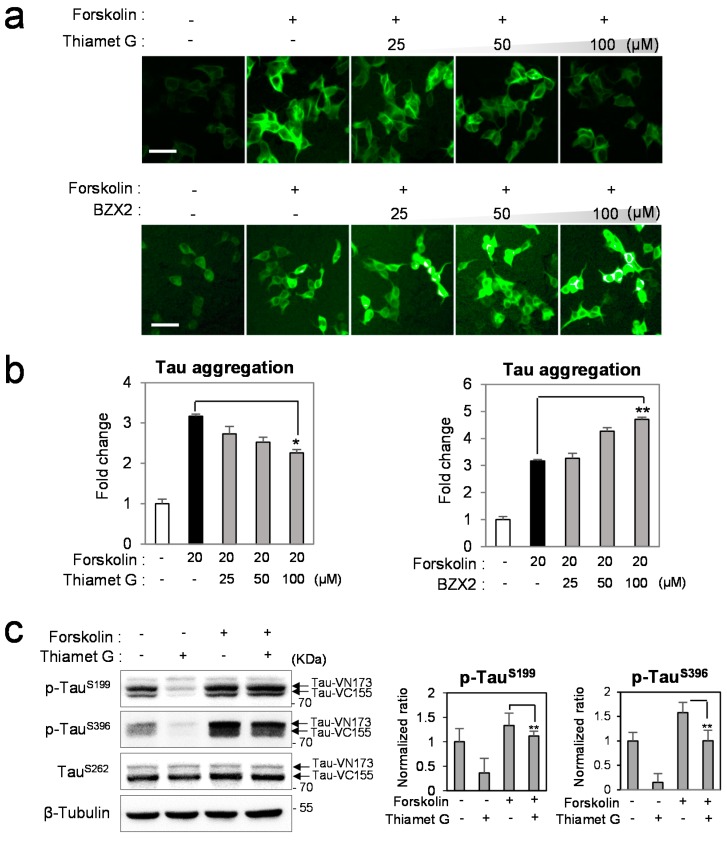

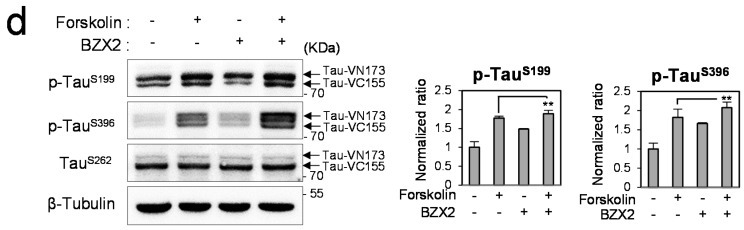
The regulatory effect of *O*-GlcNAcylation under tau hyperphosphorylation. (**a**,**b**) Tau-BiFC cells were treated with Thiamet G or BZX2 (25, 50 and 100 µM) with forskolin (20 µM) for 24 h. Then, cellular responses of tau-BiFC fluorescence were imaged by using Operetta^®^ (**a**) and quantified by Harmony 3.1 software (**b**); The tau-BiFC levels are presented as the normalized percentage of forskolin-treated cells. The significance of the experiments was determined with paired *t*-test. * *p <* 0.05, ** *p <* 0.01. Scale bar = 50 µm. (**c**,**d**) Immunoblot analysis of tau phosphorylation and the quantification. For immunoblot analysis, tau-BiFC cells were incubated with Thiamet G (100 µM, **c**), BZX2 (100 µM, **d**) or co-treated with forskolin (20 µM) for 24 h. The levels of tau phosphorylation were determined using anti-phospho-Ser199 or anti-phospho-Ser396 antibodies. Anti-β-tubulin antibody was used for loading control. The relative levels of tau phosphorylation were quantified and normalized with that of non-phosphorylated tau (TauSer262). Black arrows indicate two parts of tau-conjugated BiFC compartments (Tau-VN173 and Tau-VC155). Error bars represent standard deviation from three independent experiments. ** *p <* 0.01.

To investigate the differential regulatory effect of *O*-GlcNAc under tau phosphoylation condition, tau-BiFC cells were treated with OGA or OGT inhibitors upon the stimulation of tau phosphorylation by forskolin (Figure 2a). Forskolin, an activator of protein kinase A (PKA), is known to induce tau hyperphosphorylation [15]. Upon the treatment of forskolin (20 µM), intracellular tau aggregation was greatly induced, showing 3.2-fold increased tau-BiFC fluorescence (Figure 2b). Intracellular tau oligomerization and aggregation were confirmed by native gel analysis and immuno-fluorescence microscopy (Figure S2). Immunoblot analysis shows the increased tau phosphorylation (1.4-fold increase at Ser199 and 1.6-fold increase at Ser396) upon the treatment of Forskolin (Figure 2c,d). As expected, the forskolin-induced tau aggregation was decreased by the treatment of Thiamet G, showing a 30% decrease in tau-BiFC fluorescence response (Figure 2b). Immunoblot analysis also shows that Thiamet G decreased forskolin-induced tau phosphorylation by 21% at Ser199 and 37% at Ser396 compared with that of forkolin-treated lysates (Figure 2c). However, the reduction rate of forskolin-induced tau phosphorylation by Thiamet G was merely 21% or 37%, even at the highest concentration of Thiamet G. The low protection rate by *O*-GlcNAcylation suggests that tau has more chance to be phosphorylated, when kinase pathway is activated.

Next, we investigated dual stimulation effects on tau pathology by the co-treatment of BZX2 and forskolin (Figure 2a). When BZX2 was co-treated with forskolin, tau aggregation was facilitated more than the single treatment of either BZX2 or forskolin (Figure 2b). The result suggests that the removal of *O*-GlcNAc boosts tau phosphorylation even further, when kinases are activated. Immunoblot analysis also confirmed the increased tau phosphorylation by the co-treatment of BZX2 and forskolin (6.8% increase at Ser199 and 14% increase at Ser396) (Figure 2d). Our result clearly indicated that forskolin-induced tau aggregation is decreased by inhibiting *O*-GlcNAcase, and facilitated by inhibiting *O*-GlcNAc transferase.

### 2.3. Thiamet G Decreased BZX2-Induced Tau Aggregation

**Figure 3:**
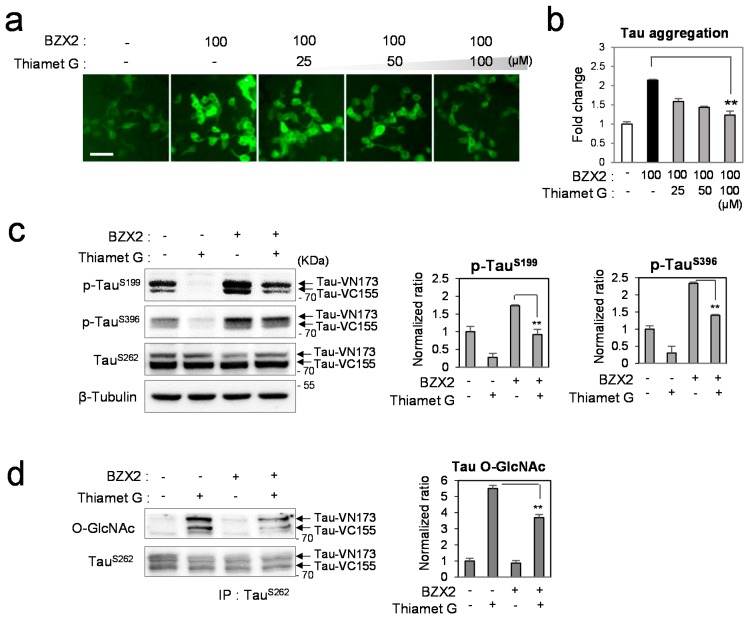
The opposite regulatory effect of OGA and OGT under co-treatment of OGA and OGT inhibitors. (**a**,**b**) Tau-BiFC cells were incubated with BZX2 (100 µM) or BZX2 co-treated with Thiamet G (25, 50 and 100 µM) for 24 h. Then, cellular responses of tau-BiFC fluorescence were imaged by using Operetta^®^ (**a**) and quantified by Harmony 3.1 software (**b**). Error bars represent the standard deviations from two independent experiments and each experiment was triplicated. The significance of the experiments was determined with paired *t*-test. ** *p* < 0.01. Scale bar = 50 µm; (**c**) Immunoblot analysis of tau phosphorylation and the quantification. For immune-blot analysis, tau-BiFC cells were incubated with Thiamet G (100 µM), BZX2 (100 µM) or Thiamet G co-treated with BZX2 for 24 h. The levels of tau phosphorylation were determined using anti-phospho-Ser199 or anti-phospho-Ser396 antibodies. Anti-β-tubulin antibody was used for loading control. The relative levels of tau phosphorylation were quantified and normalized with that of non-phosphorylated tau (TauSer262). Black arrows indicate two parts of tau-conjugated BiFC compartments (Tau-VN173 and Tau-VC155); and (**d**) Immunoblot analysis of tau *O*-GlcNAcylation levels on immuno-affinity purified tau and the quantification. The relative levels of tau *O*-GlcNAcylation were normalized with that of non-phosphorylated tau (TauSer262). Error bars represent standard deviation from three independent experiments. The significance of the experiments was determined with paired *t*-test. ** *p* < 0.01.

To scrutinize the counter-regulatory effect of OGA and OGT further, tau-BiFC cell were co-treated with Thiamet G and BZX2 (Figure 3a). As shown previously in Figure 1, BZX2 (100 µM) increases tau-BiFC fluorescence response almost two times. BZX2-induced tau aggregation decreased with co-treated Thiamet G, showing 42.5% deceased tau-BiFC response at the highest concentration (Figure 3b). Correspondingly, BZX2-induced tau phosphorylation, resulting in a 1.8-fold increase at Ser199 and 2.3-fold increase at Ser396, and reversed back to basal level by the treatment of Thiamet G (Figure 3c). These results directly suggest the opposite regulatory effect of OGA and OGT on tau aggregation and phosphorylation. Upon the treatment of Thiamet G, the level of tau *O*-GlcNAcylation was increased by 5.5-fold (Figure 3d). The increased tau *O*-GlcNAcylation efficiently decreased by the co-treated BZX2, indicating their reciprocal regulatory effect on *O*-GlcNAcylation. These results further support our conclusion above that the decrease in *O*-GlcNAcylation may have contributed to phosphorylation of tau.

### 2.4. Discussion

For many years, tau hyperphosphorylation has been believed to be the key pathological event regulating tau aggregation. Although tau phosphorylation is an important event in initiating tau pathology, recent evidence suggested that tau phosphorylation is down-stream event directly affected by tau *O*-GlcNAcylation. The hydroxyl moiety of serine and threonine residues is the modification site for phosphorylation and *O*-GlcNAcylation. Although the exact residues for *O*-GlcNAcylation remain unclear, there is a great potential that competitive modification occurs between phosphorylation and *O*-GlcNAcylation [16]. In the healthy brain, most tau proteins are protected with *O*-GlcNAc modification; therefore, cellular kinases cannot phosphorylate tau. Only after the *O*-GlcNAc is removed from tau, tau can be the substrate for cellular kinases to be phosphorylated. In this point of view, tau phosphorylation is just a secondary event, negatively regulated by *O*-GlcNAcylation [16,17]. Indeed, that impaired glucose metabolism is a common feature of neurodegenerative disease [18,19,20] has been reported. The impaired glucose metabolism often leads to depletion of UDP-GlcNAc, which is a substrate of *O*-GlcNAc transferase. Therefore, the depletion of UDP-GlcNAc decreases *O*-GlcNAc level in the brain resulting in tau phosphorylation.

On the other hand, there is a report that OGT and OGA often exist in the same protein complex with kinase and phosphatase, reinforcing their close relationship [21,22]. It was also reported that OGT and phosphatase are found in the same protein complex, which indicated that there is an enzyme complex responsible for the removal of phosphate group concomitantly adding *O*-GlcNAc moiety [23]. However, their tight regulation between the *O*-GlcNAcylation and phosphorylation remains unclear in tau research field. In this article, we have investigated the reciprocal interconnection between phosphorylation and *O*-GlcNAcylation using known inhibitors for OGA and OGT, Thiamet G and BZX2, respectively. Under basal condition, tau phosphorylation was decreased by Thiamet G and increased by BZX2, indicating the opposite regulatory effect of OGA and OGT on tau pathology. Under the tau hyperphosphorylation condition, Thiamet G did not induce a dramatic effect on tau phosphorylation, while BZX2 facilitated tau phosphorylation even further. These results indicate that when kinases are activated in cells, tau has less chance to be *O*-GlcNAcylated. Moreover, the BZX2-induced tau aggregation was efficiently decreased by Thiamet G, indicating their opposite regulatory mechanism on tau pathology.

## 3. Experimental Section

### 3.1. Cell Culture

The establishment of HEK293 tau-BiFC cell line was described in a previously published article [13]. HEK293 tau-BiFC cells were grown in Dulbecco’s modified eagle medium (DMEM) supplemented with 10% fetal bovine serum (FBS), 10,000 units/mL penicillin, 10,000 µg/mL streptomycin and 100 µg/mL Geneticin (G418) at 37 °C in a humidified atmosphere containing 5% CO2.

### 3.2. Cell Treatment and Analysis

HEK293 tau-BiFC cells were plated in a black transparent 384-well plate under the same culture conditions described above. The next day, tau-BiFC cells were treated with Thiamet G (Sigma, St. Louis, MO, USA), BZX2 or Forskolin (Sigma) at various concentrations. After the incubation, the fluorescence response in tau-BiFC cells was automatically imaged by using Operetta^®^ High Contents Image System (PerkinElmer™, Waltham, MA, USA). The intensities of tau-BiFC fluorescence were analyzed using Harmony 3.1 analysis software (PerkinElmer™). Error bars indicate standard deviation from two independent experiments. Each experiment was performed in triplicate.

### 3.3. Immunoblot Assay and Analysis

To quantify phosphorylation and *O*-GlcNAcylation level in tau-BiFC cells, immunoblot assay was performed 24 h after chemical treatment. Total cell lysates were prepared using CelLytic M (Sigma) containing protease and phosphatase inhibitor cocktail (Sigma). Ten micrograms of cell lysates were separated on 10% SDS-polyacrylamide gel and transferred to a polyvinylidene difluoride (PVDF) membrane (Millipore Co., Bedford, MA, USA). The blot was blocked with TBS-T (20 mM Tris-HCl, pH 7.6, 136 mM NaCl, and 0.15% Tween-20) containing 4% bovine serum albumin (BSA) and then incubated with primary antibody solution; anti-phospho-tau Ser199 (1:1000, ab4864), anti-phospho-tau Ser396 (1:1000, ab109390), anti-β-tubulin (1:2000, ab15568), monoclonal antibody *O*-GlcNAc (1:1000, #9875) and anti-Tau Ser262 (1:2000, ab64193) at 4 °C overnight. The blots were developed with the corresponding horseradish peroxidase (HRP)-conjugated secondary antibody and enhanced chemiluminescence HRP substrate (Thermo scientific, Hudson, NH, USA). Both bands (Tau-VN173 and Tau-VC155) intensities of the immunoblot signals from three independent experiments were quantified by ImageJ densitometry software (Version 1.48, National Institutes of Health, Bethesda, MD, USA). The average value was normalized with that of non-phosphorylated tau (TauSer262). Data are presented as normalized mean values ± standard deviation. The significance of the experiments was determined with paired *t*-test; * *p* < 0.05, ** *p* < 0.01, *** *p* < 0.001.

### 3.4. Immuno-Precipitation

Anti-tau (TauSer262) antibody was used to immunoprecipitate tau proteins from tau-BiFC cell lysates. The anti-tau (TauSer262) antibody (2 µg) was pre-incubated with 50 µL (25 µL agarose/bed volume) of protein A-sepharose beads (Sigma, P9269) for 1 hour with constant agitation at RT. The pre-incubated mixtures were gently centrifuged for 2 min and washed twice with PBS (pH 7.4). The tau-BiFC cell lysates (1 mg) were added to the pre-incubated mixtures and incubated overnight with constant agitation at 4 °C. The immunoprecipitated complexes were collected by centrifugation at 3000× *g* for 2 min at 4 °C and washed three times with 1 mL of PBS (pH 7.4). For the immunoblot analysis, immunoprecipitates were dissolved in 100 µL of Laemmli SDS sample buffer and heated for 5 min at 95 °C. Equal volume (20 µL) from all immunoprecipitated samples was loaded on 10% SDS-polyacrylamide gel.

## 4. Conclusions

In conclusion, our results indicate the protective role of *O*-GlcNAc in tau pathology and emphasize the importance of *O*-GlcNAcylation in controlling tau phosphorylation. For many years, tau phosphorylation has been considered the key mechanism initiating tau pathology. Here, we suggest the modification of the old paradigm: that tau phosphorylation is a secondary event caused by *O*-GlcNAc modification.

## Supplementary Materials

Supplementary materials can be found at http://www.mdpi.com/1422-0067/16/09/20212/s1.

## References

[1] Drechsel D.N., Hyman A.A., Cobb M.H., Kirschner M.W. (1992). Modulation of the dynamic instability of tubulin assembly by the microtubule-associated protein tau. Mol. Biol. Cell.

[2] Goode B.L., Feinstein S.C. (1994). Identification of a novel microtubule binding and assembly domain in the developmentally regulated inter-repeat region of tau. J. Cell Biol..

[3] Minoura I., Muto E. (2006). Dielectric measurement of individual microtubules using the electroorientation method. Biophys. J..

[4] Mukrasch M.D., Biernat J., von Bergen M., Griesinger C., Mandelkow E., Zweckstetter M. (2005). Sites of tau important for aggregation populate β-structure and bind to microtubules and polyanions. J. Biol. Chem..

[5] Lindwall G., Cole R.D. (1984). Phosphorylation affects the ability of tau protein to promote microtubule assembly. J. Biol. Chem..

[6] Mazanetz M.P., Fischer P.M. (2007). Untangling tau hyperphosphorylation in drug design for neurodegenerative diseases. Nat. Rev. Drug Discov..

[7] Mullane K., Williams M. (2013). Alzheimer’s therapeutics: Continued clinical failures question the validity of the amyloid hypothesis—But what lies beyond?. Biochem. Pharmacol..

[8] Liu F., Iqbal K., Grundke-Iqbal I., Hart G.W., Gong C.-X. (2004). *O*-GlcNAcylation regulates phosphorylation of tau: A mechanism involved in Alzheimer’s disease. Proc. Natl. Acad. Sci. USA.

[9] Liu F., Shi J., Tanimukai H., Gu J., Grundke-Iqbal I., Iqbal K., Gong C.-X. (2009). Reduced *O*-GlcNAcylation links lower brain glucose metabolism and tau pathology in Alzheimer’s disease. Brain.

[10] Yuzwa S.A., Macauley M.S., Heinonen J.E., Shan X., Dennis R.J., He Y., Vocadlo D.J. (2008). A potent mechanism-inspired *O*-GlcNAcase inhibitor that blocks phosphorylation of tau *in vivo*. Nat. Chem. Biol..

[11] Yuzwa S.A., Shan X., Macauley M.S., Clark T., Skorobogatko Y., Vosseller K., Vocadlo D.J. (2012). Increasing *O*-GlcNAc slows neurodegeneration and stabilizes tau against aggregation. Nat. Chem. Biol..

[12] Jiang J., Lazarus M.B., Pasquina L., Sliz P., Walker S. (2012). A neutral diphosphate mimic crosslinks the active site of human *O*-GlcNAc transferase. Nat. Chem. Biol..

[13] Tak H., Haque M.M., Kim M.J., Lee J.H., Baik J.-H., Kim Y.S., Kim D.J., Grailhe R., Kim Y.K. (2013). Bimolecular fluorescence complementation; lighting-up tau-tau interaction in living cells. PLoS ONE.

[14] Han D.H., Na H.K., Choi W.H., Lee J.H., Kim Y.K., Won C., Lee M.J. (2014). Direct cellular delivery of human proteasomes to delay tau aggregation. Nat. Commun..

[15] Liu S.J., Zhang J.Y., Li H.L., Fang Z.Y., Wang Q., Deng H.M., Wang J.Z. (2004). Tau becomes a more favorable substrate for GSK-3 when it is prephosphorylated by PKA in rat brain. J. Biol. Chem..

[16] Arnold C.S., Cole R.N., Johnson G.V.W., Hart G.W. (1994). Tau is a glycoprotein. Soc. Neurosci. Abstr..

[17] Arnold C.S., Johnson G.V., Cole R.N., Dong D.L.Y., Lee M., Hart G.W. (1996). The microtubule-associated protein tau is extensively modified with *O*-linked *N*-acetylglucosamine. J. Biol. Chem..

[18] Kalaria R.N., Harik S.I. (1989). Reduced glucose transporter at the blood-brain barrier and in cerebral cortex in Alzheimer disease. J. Neurochem..

[19] Kennedy A.M., Frackowiak R.S., Newman S.K., Bloomfield P.M., Seaward J., Roques P., Rossor M.N. (1995). Deficits in cerebral glucose metabolism demonstrated by positron emission tomography in individuals at risk of familial Alzheimer’s disease. Neurosci. Lett..

[20] Keller J.N., Pang Z., Geddes J.W., Begley J.G., Germeyer A., Waeg G., Mattson M.P. (1997). Impairment of glucose and glutamate transport and induction of mitochondrial oxidative stress and dysfunction in synaptosomes by amyloid β-peptide: Role of the lipid peroxidation product 4-hydroxynonenal. J. Neurochem..

[21] Zeidan Q., Hart G.W. (2010). The intersections between *O*-GlcNAcylation and phosphorylation: Implications for multiple signaling pathways. J. Cell Sci..

[22] Slawson C., Lakshmanan T., Knapp S., Hart G.W. (2008). A mitotic GlcNAcylation/phosphorylation signaling complex alters the posttranslational state of the cytoskeletal protein vimentin. Mol. Biol. Cell.

[23] Wells L., Kreppel L.K., Comer F.I., Wadzinski B.E., Hart G.W. (2004). *O*-GlcNAc transferase is in a functional complex with protein phosphatase 1 catalytic subunits. J. Biol. Chem..

